# Supplementary material for: Polyketide synthase gene clusters in the ericoid mycorrhizal fungus Oidiodendron maius: genome-wide prediction, expression profiling, and functional characterization of knockout mutants
Source: Front Plant Sci. 2026 Sep 16;17:1924430. doi: 10.3389/fpls.2026.1924430 (PMC13623903; doi:10.3389/fpls.2026.1924430)
Supplement: Supplementary file 1 [file SupplementaryFile1.zip › Supplementary Materials/Supplementary Folder 2.zip!/Folder S2/Oidma1_SM_Step10_scaffold_23_MultiOmics_Karyoplot.pdf]

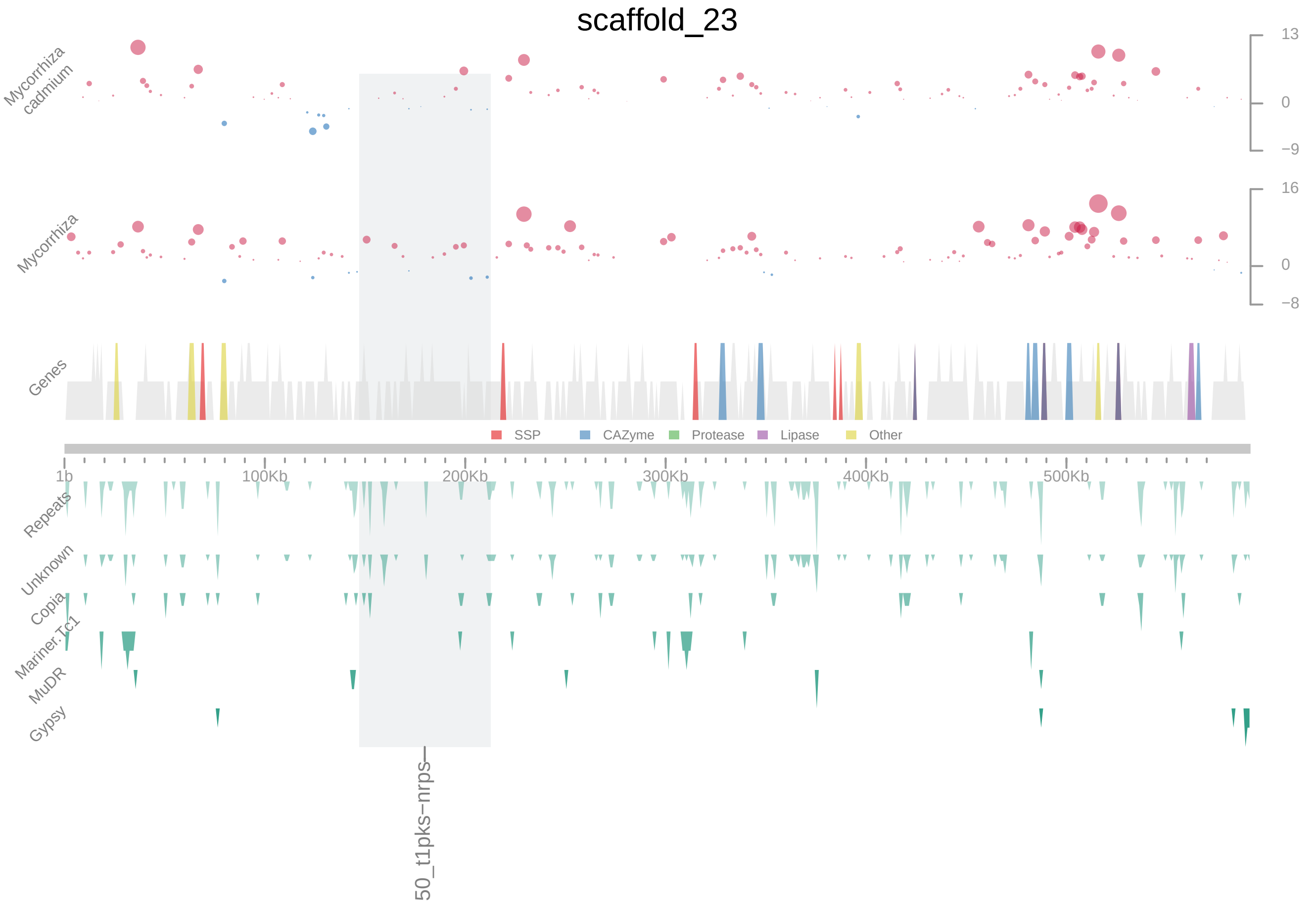
scaffold_23
Mycorrhiza cadmium
Mycorrhiza
Genes
SSP
CAZyme
Protease
Lipase
Other
13
0
−9
16
0
−8
1b
100Kb
200Kb
300Kb
400Kb
500Kb
Repeats
Unknown
Copia
Mariner.Tc1
MuDR
Gypsy
50_t1pks–nrps
